# RA-Net: reverse attention for generalizing residual learning

**DOI:** 10.1038/s41598-024-63623-6

**Published:** 2024-06-04

**Authors:** Zhenyuan Wang, Xuemei Xie, Jianxiu Yang, Xiaodan Song

**Affiliations:** 1 School of Artificial Intelligence, Xidian University, Xi’an, 710071 China; 2 Guangzhou Institute of Technology, Xidian University, Guangzhou, 510555 China; 3 Pazhou Lab, Huangpu, 510555 Guangzhou China

**Keywords:** Reverse attention, Generalized residual learning, Identity mapping, Modified global response normalization, Computer science, Computational science

## Abstract

Since residual learning was proposed, identity mapping has been widely utilized in various neural networks. The method enables information transfer without any attenuation, which plays a significant role in training deeper networks. However, interference with unhindered transmission also affects the network’s performance. Accordingly, we propose a generalized residual learning architecture called reverse attention (RA), which applies high-level semantic features to supervise low-level information in the identity mapping branch. It means that higher semantic features selectively transmit low-level information to deeper layers. In addition, we propose a Modified Global Response Normalization(M-GRN) to implement reverse attention. RA-Net is derived by embedding M-GRN in the residual learning framework. The experiments show that the RA-Net brings significant improvements over residual networks on typical computer vision tasks. For classification on ImageNet-1K, compared with resnet101, RA-Net improves the Top-1 accuracy by 1.7% with comparable parameters and computational cost. For COCO detection, on Faster R-CNN, reverse attention improves box AP by 1.9%. Meanwhile, reverse attention improves UpperNet’s mIoU by 0.7% on ADE20K segmentation.

## Introduction

**Figure 1:**
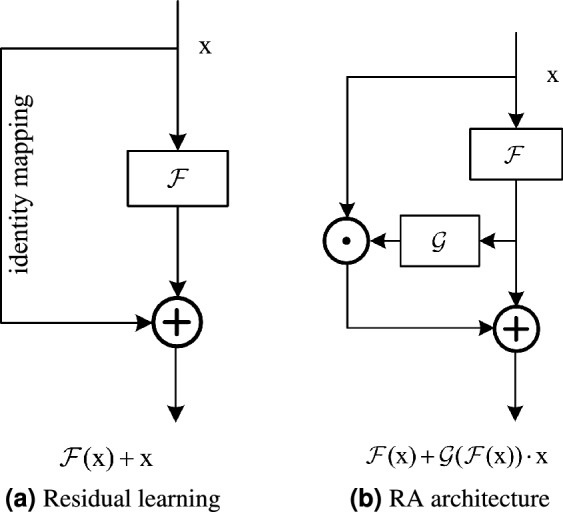
Comparison of residual learning and the proposed reverse attention (RA) architecture. (**a**) Residual learning architecture in ResNet^2^. (**b**) Our proposed generalized architecture. The approach of scaling $\text{x}$ with high-semantic ${{\mathscr {G}}}({{\mathscr {F}}}({\mathop {\text {x}}\nolimits } ))$ is called reverse attention.

Convolutional neural networks (CNNs) and Transformers are two dominant frameworks in modern computer visual encoding systems. Similarly, they both apply identity mapping (skip connection) in their unit blocks. Identity mapping^1^, as shown in Fig. 1a, also known as shortcut connection, was proposed in residual learning networks (ResNets)^2^. The unhindered transmission of information allows backpropagation to optimize extremely deep networks. Thus the architecture of residual learning is widely adopted in various state-of-the-art networks. For instance, the identity mapping is utilized twice in the MetaFormer’s architecture^3^.

To enhance the model’s capacity, numerous approaches are devoted to studying the structure of ${{{\mathscr {F}}}}$ in Fig. 1. For example, ConvNeXt^4^ fits ${{{\mathscr {F}}}}$ through a pure CNNs structure, while Vision Transformer (ViT)^5^ adopts a self-attention mechanism. These models all adopt residual learning methods. The shortcut connection seems impeccable. However, we show that there exists a more general architecture. In vision-related tasks^6^, there is a lot of interference. For example, the background of the image affects the recognition of the target. Transmission of information indiscriminately causes interference to accumulate continuously to deeper layers, thus affecting network performance. We are committed to exploring a new architecture that maintains the advantages of residual learning while weakening its drawbacks. Therefore, a generalized residual learning architecture is proposed, which is the reverse attention (RA) mechanism. It leverages high-level semantics to supervise relatively low-level information in reverse.

Currently, it is the self-attention mechanism^7^ that is favored by the computer vision community. Self-attention is mutual supervision among tokens at the same semantic level. For example, all patches in ViT^5^ undergo the same processing before attention calculation, and they all have the same receptive field and depth. Unlike self-attention, RA uses high-level semantics to supervise relatively low-level semantics (The ‘levels’ of semantics can be enriched by the number of stacked layers (depth)). As shown in Fig. 1b, ${{\mathscr {F}}}\mathrm{{(x)}}$ has higher semantics than $\mathrm{{x}}$. This semantically dominant, cross-level attention mechanism adaptively determines whether it is necessary to transmit low-level information to deeper layers.

The introduction of reverse attention increases the dynamics of the model. In addition to studying the structure of ${{\mathscr {F}}}$, we also provide a new idea to improve the model’s capacity, which is to optimize the reverse attention branch ${{\mathscr {G}}}$ in Fig. 1b. It is the fundamental component of the reverse attention architecture. A poorly constructed ${{\mathscr {G}}}$ does not enhance the model’s performance or even degrades it. To demonstrate the effectiveness of reverse attention and reduce additional parameters, we implement ${{\mathscr {G}}}$ with Modified Global Response Normalization (M-GRN). Furthermore, when ${\mathscr {F}}\mathrm{{(x)=0}}$, ${{{\mathscr {G}}}({{\mathscr {F}}}(\mathrm{{x}})) = {{\mathscr {G}}}(0) = 1}$, which complies with the original intention of residual learning^2^. Therefore, the reverse attention architecture is a generalized form of residual learning.

Our main contributions are summarized as follows:

- Reverse attention mechanism is proposed. It utilizes high-level semantics to supervise relatively low-level semantics. The existence of a semantic gap enhances the effectiveness of reverse attention.
- With the help of reverse attention, we propose the RA architecture, which is a generalized form of residual learning. It proves that performance can also be improved by optimizing the architecture.
- We propose Modified Global Response Normalization (M-GRN) to implement RA architecture, which introduces negligible extra parameters.
- RA-Net is derived from the RA architecture. Experiments prove that RA-Net brings significant improvements over residual networks.

## Related work

### Residual learning

Residual learning can be traced back to the proposal of ResNet^2^. As deeper networks begin to converge, a problem is revealed: as the depth of the network increases, the accuracy becomes saturated and then decreases rapidly. ResNet addresses the degradation problem by adding an identity mapping branch. Rather than expecting each stacked layer to fit directly into the desired underlying mapping, ResNet explicitly makes these layers fit into the residual mapping. In extreme cases, if an identity mapping is optimal, it is much easier to push the residuals to zero than to fit an identity mapping with multiple layers. Residual learning is favored by the computer vision community and is present in almost all advanced frameworks. Pure CNNs frameworks such as ConvNeXt^4,8^, ParC-Net^9^, RepLKNet^10^, PoolFormer^3^, MobileNet^11^, ShuffleNet^12^ and EfficientNet^13^ focus on different aspects of accuracy and efficiency. Another category is attention-based frameworks such as ViT^5^, Swin Transformer^14^, and PVT^15^. The hybrid structure is also a hot spot of current research, such as Mobile-Former^16^, CoaT^17^, and Mobile-VIT^18^. Without exception, these approaches all rely on the identity mapping branch to optimize deeper networks.

### Attention mechanism

Attention mechanism plays a crucial role in computer vision tasks, especially self-attention in transformer. ViT^5^ directly applied to image patch sequences for image classification tasks. Meanwhile, more attention-based models are applied to computer vision tasks such as detection^19^ and segmentation. Self-attention is applied to establish relationships among patches. And all patches perform the same operation before the self-attention calculation, thus they have the same semantic level. Besides transformers, there are other types of attention mechanisms. SE-Net^20^ is proposed to re-estimate the channel responses of convolutional features. And it belongs to channel-wise feature supervision. Based on SE-Net, CBAM^21^ adds a spatial attention module. These are plug-and-play attention methods with great flexibility. Different from the above attention methods, what we propose is a reverse attention mechanism, that is, the high-level semantics are used to supervise the low-level semantics in reverse. It is more in line with the human learning patterns in which teachers with more experience instruct students. Other attention mechanisms rarely consider semantic level issues.

### General architecture

Transformers show great potential in computer vision tasks. It is widely believed that their attention-based token mixer module contributes the most to their capabilities^22^. However, MetaFormer^3^ proves that the general architecture of the Transformer is more important to the model’s performance. The MetaFormer derived model PoolFormer, utilizing a pooling-based token mixer, surprisingly achieves competitive performance on several computer vision tasks. This suggests the importance of architecture in neural networks. Therefore, it is more attractive to explore the impact of general architecture on performance. Deeply inspired by MetaFormer, reverse attention is applied to generalize the residual learning framework. We prioritize leveraging the strengths of the architecture itself to improve the performance. Reverse attention is implemented by M-GRN, which introduces negligible parameters and computation consumption. Consequently, the performance improvements achieved through reverse attention can be primarily attributed to the inherent advantages of its architecture.

## Methods

### RA architecture

Residual learning (identity mapping) is the dominant architecture in current models. The formulation of residual learning is as follows:

1 $$\begin{aligned} \mathrm{{y}} = {{\mathscr {F}}}\mathrm{{(x)}} + \mathrm{{x}}, \end{aligned}$$

where $\text{x}$ is the identity mapping branch, which represents shallow layer information that is added directly to the higher-level semantic ${{\mathscr {F}}}\mathrm{{(x)}}$ without hindrance. The indiscriminate transmission of information also leads to the accumulation of interference to deeper layers. Therefore, we propose the reverse attention architecture, which generalizes the residual learning. Reverse attention, as the name implies, is the opposite of the forward process. It is an attention mechanism that utilizes high-semantic features to supervise low-semantic information. The generalized equation is:

2 $$\begin{aligned} \mathrm{{y}} = {{\mathscr {F}}}({\mathop {\text {x}}\nolimits } ) + {{\mathscr {G}}}({\mathscr {F}} ({\mathop {\text {x}}\nolimits } )) \cdot {\mathop {\text {x}}\nolimits }, \end{aligned}$$

where the ${{\mathscr {G}}}({{\mathscr {F}}}({\mathop {\text {x}}\nolimits } ))$ implements reverse attention. ${{\mathscr {F}}}$ contains some convolutional layers to aggregate information of spatial and channel dimensions, which is used to improve the receptive field and capacity of the network. In general, the semantic “level” can be enriched by the number of stacked layers (depth)^2^. Therefore, compared with $\text{x}$, ${{\mathscr {F}}}\mathrm{{(x)}}$ has a higher semantic level, which ensures that it contains richer information. The semantically dominant, cross-level attention mechanism adaptively determines the degree to which $\text{x}$ flows to the deeper layer. The model adaptively retains valuable information while blocking interference. In particular, the RA architecture retains the advantages of residual learning. When the condition ${{\mathscr {G}}}({\mathscr {F}}({\mathop {\text {x}}\nolimits }))=1$ is satisfied, Eq. (2) is equivalent to Eq. (1), i.e., the RA architecture degenerates to the residual learning approach. This is the key reason why residual learning can be generalized through reverse attention.

Nowadays, the differences among the different models are mainly in the ${{\mathscr {F}}}$, that is, by optimizing the design of ${{\mathscr {F}}}$ to increase the model capacity. In response to this limitation, the RA architecture provides another way. The branch ${{\mathscr {G}}}$ takes ${{\mathscr {F}}}\mathrm{{(x)}}$ as input and its output is directly multiplied by $\text{x}$. Fitting ${{\mathscr {G}}}$ with different methods directly affects the model’s performance. In the following subsection, we introduce the RA block derived from the RA architecture, including a simple instantiation of ${{\mathscr {G}}}$.

**Figure 2:**
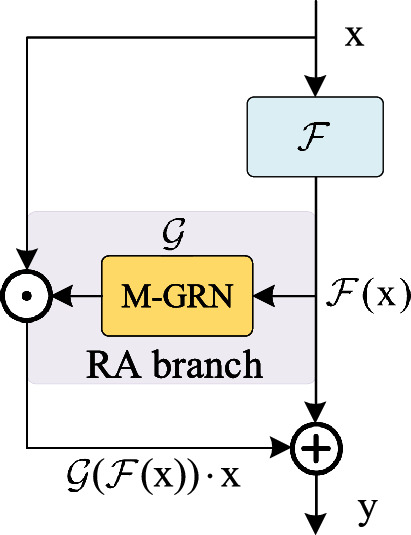
Illustration of the RA block. M-GRN represents Modified Global Response Normalization.

### RA block

**Figure 3:**
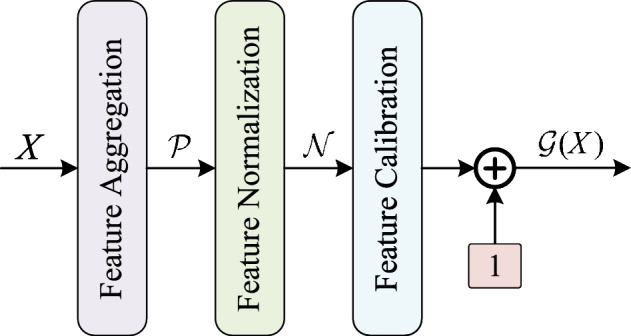
Illustration of RA branch instantiation.

We present the RA block derived from the RA architecture. As shown in Fig. 2, regarding the optimization of ${{\mathscr {F}}}$, there are many related literatures for a comprehensive analysis. Therefore, this paper focuses on the design of the reverse attention branch. Two conditions need to be satisfied for the reverse attention branch. The first one is to ensure that ${{\mathscr {G}}}({\mathscr {F}}({\mathop {\text {x}}\nolimits }))=1$ when ${{\mathscr {F}}}\mathrm{{(x)=0}}$, which means ${{\mathscr {G}}}(0) = 1$. It aims to preserve the advantages of residual learning^2^. The other one is to ensure that the semantic level of ${{\mathscr {F}}}\mathrm{{(x)}}$ is higher than $\text{x}$. The two commonly used attention functions, Sigmoid^23^ and Softmax^24^, no longer satisfy the first condition. Therefore, we propose a novel implementation of the RA branch (${{\mathscr {G}}}$) based on M-GRN. As shown in Fig. 3, given an input feature, $X \in {R^{H \times W \times C}}$, M-GRN consists of three steps: (1) global feature aggregation, (2) feature normalization and (3) feature calibration.

To obtain global features and reduce extra computational costs (FLOPs), the feature spatial dimension ($H \times W$) is compressed at the beginning of the RA branch. There are many ways to obtain global features, such as Global Average Pooling (GAP), Global Max Pooling (GMP), L1-Norm, and L2-Norm. We choose the optimal method L2-Norm through comparative experiments. The equation is as follows:

3 $$\begin{aligned} {{\mathscr {P}}}(X) = \{ \left\| {{X_1}} \right\| ,\left\| {{X_i}} \right\| , \ldots ,\left\| {{X_C}} \right\| \} \in {R^C}, \end{aligned}$$

where ${{\mathscr {P}}}{(X)_i} = \left\| {{X_i}} \right\|$ is a scalar, aggregate the information of the *i*-th channel. *C* represents the number of feature channels. And $\left\| \cdot \right\|$ is L2-Norm.

Regarding the normalization function, the most commonly used is standardization, such as BN^25^ and LN^26^. Its equation is as follows:

4 $$\begin{aligned} {{\mathscr {N}}}(\left\| {{X_i}} \right\| ) = \frac{{\left\| {{X_i}} \right\| - \mu }}{{\sigma + \varepsilon }}\in R, \end{aligned}$$

where $\varepsilon$ is a small float added to the denominator to avoid dividing by zero. This approach does not satisfy the first condition of reverse attention. When ${{\mathscr {P}}}{(X_i)} = \left\| {{X_i}} \right\| = 0$, ${{\mathscr {N}}}(\left\| {{X_i}} \right\| )={{ - \mu } {(\sigma + \varepsilon )}}$. GRN^8^ provides another efficient normalization method. The aggregated values are normalized as follows:

5 $$\begin{aligned} {{\mathscr {N}}}(\left\| {{X_i}} \right\| ) = \frac{{\left\| {{X_i}} \right\| }}{{\frac{1}{C}\sum \nolimits _{j = 1, \ldots ,C} {\left\| {{X_j}} \right\| + \varepsilon } }} \in R. \end{aligned}$$

When $\left\| {{X_i}} \right\|$ is equal to zero, the value of ${{\mathscr {N}}}(\left\| {{X_i}} \right\| )$ is also zero. Compared to Eq. (4), the normalization method of Eq. (5) is easier to modify to satisfy the reverse attention condition. It is described in detail in Step 3.

To facilitate optimization, two learnable parameters, $\gamma$ and $\beta$, are usually introduced to calibrate the features. The formula is as follows:

6 $$\begin{aligned} {{\mathscr {G}}}(X) = \gamma \cdot {{\mathscr {N}}}({{\mathscr {P}}}(X)) + \beta \in {R^C}. \end{aligned}$$

The method cannot be directly applied in the reverse attention branch. We first remove the bias term $\beta$ in Eq. (6) and add a constant 1. Equation (6) is modified as:

7 $$\begin{aligned} {{\mathscr {G}}}(X) = \gamma \cdot {{\mathscr {N}}}({{\mathscr {P}}}(X)) + 1\in {R^C}. \end{aligned}$$

It is easy to conclude that ${{\mathscr {G}}}(0) = 1$. In addition, we only introduce $\gamma$ as a learnable parameter, which adds insignificant parameters and computational cost. Therefore, it can effectively verify the performance of RA architecture.

It is worth mentioning how to ensure that the semantic-level of ${{\mathscr {F}}}(x)$ is higher than that of *x*, which is the second condition that the RA block needs to satisfy. ResNet^2^ shows that the “level” of semantics can be enriched by stacking layers (depth). However, the semantic gap does not exist for untrained models with randomly initialized parameters. Therefore, ${{\mathscr {F}}}$ should be trained preferentially compared to the RA branch. We adopt two approaches to make ${{\mathscr {F}}}(x)$ be trained preferentially. Firstly, we initialize $\gamma$ to 0, which means ${{\mathscr {G}}}(X) = 1$. This setup allows the RA block to initially perform residual learning and gradually adapt during training. Furthermore, we modify Eq. (7) as follows:

8 $$\begin{aligned} {{\mathscr {G}}}(X) = \frac{{\gamma \cdot {{\mathscr {N}}}({{\mathscr {P}}}(X))}}{temp } + 1\in {R^C}, \end{aligned}$$

where *temp* represents temperature annealing strategy^27^ for facilitating the training process. The *temp* is not a fixed value but changes dynamically with training iterations. In this paper, we linearly reduce *temp* from 30 to 1 in the first 10 epochs of training. It slows down the training of ${{\mathscr {G}}}$, widening the semantic gap between ${{\mathscr {F}}}(x)$ and *x*.

As a generalized architecture for residual learning, we first consider applying reverse attention to ResNet^2^. We maintain all parts in ResNet and only embed the reverse attention branch, which is called RA-ResNet. We further validate the reverse attention mechanism on lightweight models, such as MobileNetV2^11^. It is worth noting that mobileNetV2 contains two different unit blocks, which differ in whether skip connections (identity mappings) are included. We only embed RA branches in blocks containing skip connections, named RA-MobileNetV2. Experiments are carried out mainly on these two types of models to comprehensively verify the performance of the reverse attention mechanism.

## Experiments

### Image classification

#### Datasets

ImageNet-1K^28^ is one of the most classic classification datasets. It contains about 1.3 M training images and 50 K validation images, covering rich scenes and common 1k categories. Therefore, it can accurately represent the difference in accuracy of different methods. Consistent with most approaches, the performance is evaluated by top-1 and top-5 recognition rates on the ImageNet-1K validation set. More training details are listed in Table 1.

**Table 1:** Configuration for training on ImageNet-1K.

Config	RA-ResNet			RA-MobileNetV2		
	18	50	101	0.5	0.75	1.0
Weight init	kaiming.normal					
Optimizer	SGD					
Learning rate	0.1			0.05		
momentum	0.9					
Weight decay	0.0001			0.00004		
Batch size	256					
Training epochs	100			150		
Dropout rate	0	0.2	0.2	0	0.1	0.2
Temperature annealing strategy	True					
Ema^29^	None					
Data augment	random crop, horizontal flip					
Framework	PyTorch^30^					
Hardware	2 RTX4090 GPUs					

#### CNN backbones

The MobileNetV2^11^ and ResNet^2^ families are selected for experimentation with both lightweight and large CNN architectures. In particular, we choose six backbones, including ResNet18, ResNet50, ResNet101, and MobileNetV2($1.0\times$, $0.75\times$, $0.5\times$). These backbones are utilized to verify the effect of the reverse attention mechanism on models with different sizes and depths.

**Table 2:** Comparison of proposed RA with SE, CBAM in ResNet18, ResNet50, Resnet101 backbones.

Models	Size	Param.(M)	FLOPs(G)	Top-1 Acc.	Top-5 Acc.	Training hours
ResNet18	$224 ^2$	11.69	1.824	70.2	89.4	12.4
+SE	$224 ^2$	11.78 ($\uparrow$0.09)	1.824	71.0 ($\uparrow$0.8)	90.0 ($\uparrow$0.6)	12.6
+CBAM	$224 ^2$	11.87 ($\uparrow$0.18)	1.825	71.1 ($\uparrow$0.9)	89.9 ($\uparrow$0.5)	13.2
**+RA**	$224 ^2$	11.69 ($\uparrow$0.00)	1.824	**71.2 (**$\uparrow$**1.0)**	**90.1 (**$\uparrow$**0.7)**	12.7
ResNet50	$224 ^2$	25.56	4.134	76.2	93.0	28.9
+SE	$224 ^2$	28.06 ($\uparrow$2.50)	4.136	77.3 ($\uparrow$1.1)	93.6 ($\uparrow$0.6)	35.8
+CBAM	$224 ^2$	30.57 ($\uparrow$5.01)	4.140	77.4 ($\uparrow$1.2)	93.7 ($\uparrow$0.7)	44.3
**+RA**	$224 ^2$	25.56 ($\uparrow$0.00)	4.134	**77.6 (**$\uparrow$**1.4)**	**93.8 (**$\uparrow$**0.8)**	36.5
ResNet101	$224 ^2$	44.55	7.866	77.4	93.7	46.8
+SE	$224 ^2$	49.33 ($\uparrow$4.78)	7.871	78.4 ($\uparrow$1.0)	94.1 ($\uparrow$0.4)	57.2
+CBAM	$224 ^2$	54.11 ($\uparrow$9.56)	7.878	78.5 ($\uparrow$1.1)	94.2 ($\uparrow$0.5)	76.5
**+RA**	$224 ^2$	44.58 ($\uparrow$0.03)	7.866	**79.1 (**$\uparrow$**1.7)**	**94.3 (**$\uparrow$**0.6)**	59.8

The experimental results are obtained by training 100 epochs on ImageNet-1K. FLOPs are obtained when the input image is ${224\times 224}$. “Training hours” is evaluated on 2 RTX4090 GPUs.

Significant values are in bold.

#### Results comparison with ResNets

We first implement experiments on ResNets^2^. The results are shown in Table 2. All these models are trained only on the ImageNet-1K training set and report their accuracy on the validation set. We mainly compare RA-Net with ResNet, SE-Net, and CBAM. As shown in Table 2, we analyze the results from the following perspectives, including parameters, FLOPs, Top-1, and Top-5 accuracy. FLOPs are obtained when the input size is ${224 \times 224}$. When ResNet18 is selected as the baseline, RA-ResNet18 improves the Top-1 accuracy by 1.0% with comparable parameters and FLOPs. Meanwhile, the Top-1 accuracy of SE-ResNet18 and CBAM-ResNet18 is improved by 0.8% and 0.9%, respectively. Similarly, it can be seen that when ResNet50 is the baseline, the Top-1 accuracy of SE-ResNet50, CBAM-ResNet50, and RA-ResNet50 are increased by 1.1%, 1.2%, and 1.4%, respectively. There is only a slight difference in the FLOPs of RA, SE, and CBAM due to the use of feature aggregation.

**Table 3:** Comparison of proposed RA with SE, CBAM in MobileNetV2 backbones.

Models	Size	Param. (M)	FLOPs (G)	Top-1 Acc.	Top-5 Acc.	Training hours
MobileNetV2 (0.5$\times$)	$224 ^2$	1.969	111.7	64.3	85.2	18.8
+SE	$224 ^2$	1.972 ($\uparrow$0.003)	111.7	65.1 ($\uparrow$0.8)	85.7 ($\uparrow$0.5)	18.9
+CBAM	$224 ^2$	1.976 ($\uparrow$0.007)	112.3	65.3 ($\uparrow$1.0)	86.1 ($\uparrow$0.9)	19.2
**+RA**	$224 ^2$	1.969 ($\uparrow$0.000)	111.7	**65.8 (**$\uparrow$**1.5)**	**86.5 (**$\uparrow$**1.3)**	18.9
MobileNetV2 (0.75$\times$)	$224 ^2$	2.636	232.5	69.2	88.8	19.8
+SE	$224 ^2$	2.643 ($\uparrow$0.007)	232.5	70.0 ($\uparrow$0.8)	89.4 ($\uparrow$0.6)	20.2
+CBAM	$224 ^2$	2.650 ($\uparrow$0.014)	233.1	70.3 ($\uparrow$1.1)	89.4 ($\uparrow$0.6)	21.3
**+RA**	$224 ^2$	2.636 ($\uparrow$0.000)	232.5	**70.4 (**$\uparrow$**1.2)**	**89.5 (**$\uparrow$**0.7)**	20.3
MobileNetV2 (1.0$\times$)	$224 ^2$	3.505	327.5	71.6	90.2	21.9
+SE	$224 ^2$	3.516 ($\uparrow$0.011)	327.5	72.1 ($\uparrow$0.5)	90.8 ($\uparrow$0.6)	22.3
+CBAM	$224 ^2$	3.529 ($\uparrow$0.024)	328.1	72.4 ($\uparrow$0.8)	90.9 ($\uparrow$0.7)	23.8
**+RA**	$224 ^2$	3.505 ($\uparrow$0.000)	327.5	**72.5 (**$\uparrow$**0.9)**	**90.9 (**$\uparrow$**0.7)**	23.4

The experimental results are obtained by training 150 epochs on ImageNet-1K. FLOPs are obtained when the input image is ${224\times 224}$. “Training hours” is evaluated on 2 RTX4090 GPUs.

Significant values are in bold.

To verify the performance of the reverse attention mechanism on a deeper network, we further conduct comparative experiments on ResNet101. The proposed RA-ResNet101 shows a significant performance improvement compared to SE and CBMA. As shown in Table 2, embedding SE and CBAM in ResNet101 increases the parameters of the model by 4.78 M and 9.56 M. And their Top-1 accuracy increased by 1.0% and 1.1%. Meanwhile, the RA branch embedded in ResNet101 leads to a 1.7% improvement in Top-1 accuracy with only 0.03M additional parameters.

Overall, compared to SE and CBAM, RA utilizes fewer extra parameters to bring greater performance improvements. In addition, with the increase of network depth, the advantage of reverse attention becomes obvious. For example, introducing SE in ResNet18, ResNet50 and ResNet101 increases the Top-1 accuracy by 0.8%, 1.1%, and 1.0%. Similarly, CBAM improves the performance by 0.9%, 1.2% and 1.1%. The improvement stays around 1.0%. On the contrary, RA improves the performance of ResNet18, ResNet50, and ResNet101 by 1.0%, 1.4%, and 1.7%. It shows an upward trend. We infer that the reverse attention mechanism is more conducive to the optimization of deeper networks without compromising the advantages of residual learning. Meanwhile, comparing the training time of these methods, we find that the “Training hours” of RA and SE are close, slightly higher than the baseline. CBAM’s “Training hours” is significantly larger than other methods, which is caused by its simultaneous use of spatial and channel attention.

#### Results comparison with MobileNetV2

We further verify the performance of the reverse attention mechanism on the lightweight model MobileNetV2. The experiment results are shown in Table 3. Overall, RA, SE, and CBAM all improve the performance. RA has the highest accuracy when the parameters and FLOPs are comparable to the baseline. For example, when selecting MobileNetV2 (0.5$\times$) as the baseline, SE, CBAM, and RA improve the Top-1 accuracy by 0.8%, 1.0%, and 1.5%. For MobileNetV2 (0.75$\times$), embedding SE, CBAM and RA into the baseline, the Top-1 accuracy improvement is 0.8%, 1.1% and 1.2%. Similarly, SE, CBAM, and RA improve the Top-1 accuracy on MobileNetV2 (1.0$\times$) by 0.5%, 0.8% and 0.9%. Therefore, we can conclude that the reverse attention mechanism is also effective for lightweight models. Furthermore, we find that the training time (“Training hours”) of these methods is at the same level, due to the fact that the baseline MobileNetV2 is a lightweight model.

### Downstream tasks

#### Datasets

We evaluate the performance of the reverse attention mechanism in downstream tasks on the COCO^31^ and ADE20K^32^ datasets. Following standard training and testing protocols, trainval35k set and minimal set (5 K images) are utilized for training and testing. Consistent with most detectors^33^, the performance is evaluated by Average Precision (AP)^31^.

#### Detection results

The performances of SE, CBAM, and RA are compared on two classical detectors Faster R-CNN and Mask R-CNN. ResNet50 is adopted as the backbone of the detector. The experimental results of object detection are shown in Table 4. For example, when using ResNet50 as the backbone of the Faster R-CNN, RA increased the box AP by 1.9%. However, SE and CBAM increased the box AP by 1.6% and 1.5% when adding 2.51M and 5.01M parameters, respectively. On the Mask R-CNN detector, SE, CBAM, and RA improve Box AP by 1.4%, 1.4%, and 1.7%.

**Table 4:** Results of object detection on the COCO dataset.

Backbone	Param.	FLOPs	$\mathrm{{AP}^{\mathrm{{box}}}}$(%)	$\mathrm{{AP}}_{50}^{\mathrm{{box}}}$(%)	$\mathrm{{AP}}_\mathrm{{S}}^{\mathrm{{box}}}$(%)	$\mathrm{{AP}}_\mathrm{{M}}^{\mathrm{{box}}}$(%)	$\mathrm{{AP}}_\mathrm{{L}}^{\mathrm{{box}}}$(%)
ResNet50 + Faster R-CNN							
Baseline	43.58 M	207.07 G	37.3	58.0	21.0	41.0	48.3
+SE	46.08 M	207.07 G	38.9 ($\uparrow$1.6)	60.2 ($\uparrow$2.2)	22.9 ($\uparrow$1.9)	43.0 ($\uparrow$2.0)	**50.2 (**$\uparrow$**1.9)**
+CBAM	48.59 M	207.10 G	38.8 ($\uparrow$1.5)	60.0 ($\uparrow$2.0)	23.3 ($\uparrow$2.3)	42.9 ($\uparrow$1.9)	49.2 ($\uparrow$0.9)
**+RA**	43.59 M	207.07 G	**39.2 (**$\uparrow$**1.9)**	**60.4 (**$\uparrow$**2.4)**	**23.5 (**$\uparrow$**2.5)**	**43.1 (**$\uparrow$**2.1)**	49.6 ($\uparrow$1.3)
ResNet50 + Mask R-CNN							
Baseline	46.22 M	260.14 G	38.2	58.7	21.8	41.8	49.5
+SE	48.73 M	260.14 G	39.6 ($\uparrow$1.4)	60.7 ($\uparrow$2.0)	23.0 ($\uparrow$1.2)	43.6 ($\uparrow$1.8)	**51.9 (**$\uparrow$**2.4)**
+CBAM	51.23 M	260.17 G	39.6 ($\uparrow$1.4)	60.4 ($\uparrow$1.7)	23.5 ($\uparrow$1.7)	43.5 ($\uparrow$1.7)	50.4 ($\uparrow$0.9)
**+RA**	46.23 M	260.14 G	**39.9 (**$\uparrow$**1.7)**	**61.1 (**$\uparrow$**2.4)**	**24.1 (**$\uparrow$**2.3)**	**43.7 (**$\uparrow$**1.9)**	50.9 ($\uparrow$1.4)

ResNet50 is adopted as the backbone. The final model weights pre-trained in ImageNet-1K are used as the initialization of the detector. FLOPs are obtained when the input image is ${1280\times 800}$.

Significant values are in bold.

#### Segmentation results

We further verify the performance of the reverse attention mechanism on the segmentation task. The results are presented in Table 5. For instance segmentation on Mask R-CNN^35^, RA improves Mask AP by 1.6%. For semantic segmentation on UperNet^36^, RA improves mIoU by 0.7%. Obviously, RA brings significant performance improvement.

**Table 5:** Results of instance and semantic segmentation on COCO and ADE20K datasets.

Backbone	$\mathrm{{AP}^{\mathrm{{mask}}}}$	Backbone	mIoU
Mask R-CNN		UperNet	
Baseline	34.6	Baseline	40.7
+SE	35.7 ($\uparrow$1.1)	+SE	40.8 ($\uparrow$0.1)
+CBAM	35.7 ($\uparrow$1.1)	+CBAM	41.0 ($\uparrow$0.3)
**+RA**	**36.2 (**$\uparrow$**1.6)**	**+RA**	**41.4 (**$\uparrow$**0.7)**

ResNet50 is adopted as the backbone of Mask R-CNN and UperNet^36^.

Significant values are in bold.

### Ablation studies

#### Feature aggregation

The purpose of the feature aggregation step is to obtain global features while reducing the cost of computation. We compare the performance of several common feature aggregation methods, including Global Max Pooling (GMP), Global Average Pooling (GAP), L1-Norm, and L2-Norm.

**Table 6:** Comparison of different feature aggregation approaches.

Method	Top-1 Acc.	Top-5 Acc.
Baseline	70.2	89.4
GMP^†^	70.3	89.5
GAP^†^	70.5	89.8
L1-Norm	71.0	89.9
**L2-Norm**	**71.2**	**90.1**

^†^Means to take the absolute value. ResNet18 is adopted as the baseline.

Significant values are in bold.

When directly adopting GMP and GAP for feature aggregation, the training of the model is unstable. However, the model converges stably using their absolute values (GMP$\dag$, GAP$\dag$). Compared with the baseline, both GMP$\dag$ and GAP$\dag$ can improve the model accuracy. For example, the Top-1 accuracy of GAP$\dag$ improves by 0.3% compared to the baseline. Additionally, we conduct experiments on L1-Norm and L2-Norm feature aggregation methods. The experimental results in Table 6 demonstrate that L2-Norm yields the optimal performance. Compared with the baseline, L2-Norm improves the Top-1 accuracy and Top-5 accuracy by 1.0 and 0.7%. Therefore, in the reverse attention branch, we implement the feature aggregation step with L2-Norm.

#### Feature normalization

Feature normalization effectively accelerates the convergence of the model. Therefore, we enumerate the commonly utilized normalization methods BN and LN and explore their effects on reverse attention. The experimental results of different feature normalization methods are reported in Table 7.

**Table 7:** Comparison of different feature normalization approaches.

Method	Top-1 Acc.	Top-5 Acc.
Baseline	70.2	89.4
None	70.1	89.4
BN	70.7	89.6
LN	71.1	89.8
**M-GRN**	**71.2**	**90.1**

“None” indicates no normalization step. ResNet18 is adopted as the baseline.

Significant values are in bold.

To verify the importance of the feature normalization step, we first conduct experiments without applying any normalization method. The absence of normalization degrades the performance. However, the addition of normalization significantly improves the performance. For instance, the utilization of BN and LN resulted in a 0.5% and 0.9% improvement in Top-1 accuracy. M-GRN uses the normalization method of Eq. (5). Compared with BN and LN, its Top-1 accuracy is increased by 0.5% and 0.1%, respectively.

#### Activation function

We explore the effect of activation functions on reverse attention performance. The experimental results are shown in Table 8.

**Table 8:** Comparison of different activation functions.

Method	Top-1 Acc.	Top-5 Acc.
Baseline	70.2	89.4
Softmax ($\frac{{\gamma \cdot {{\mathscr {N}}}({{\mathscr {P}}}(X))}}{{temp}}$)	69.9	89.2
Sigmoid ($\frac{{\gamma \cdot {{\mathscr {N}}}({{\mathscr {P}}}(X))}}{{temp}}$)	70.9	89.8
1 + $\frac{{\gamma \cdot {{\mathscr {N}}}({{\mathscr {P}}}(X))}}{{temp}}$	**71.2**	**90.1**

ResNet18 is adopted as the baseline.

Significant values are in bold.

The activation function Softmax almost cuts off the identity mapping in residual learning, so it degrades the model performance. Compared to the baseline, using Softmax in the reverse attention branch leads to a drop of 0.3% and 0.2% in Top-1 and Top-5 accuracy, respectively. The Sigmoid activation function brings a 0.7% improvement in Top-1 accuracy. In M-GRN, a constant term of 1 is added to ensure that the RA architecture degenerates into residual learning when ${\gamma \cdot {{\mathscr {N}}}({{\mathscr {P}}}(X))=0}$. The experimental results in Table 8 prove its effectiveness. Without adding extra consumption, it improves both Top-1 accuracy by 1.0% compared to the baseline.

#### Temperature annealing

The temperature annealing strategy enables the network to learn ${{\mathscr {F}}}\mathrm{{(x)}}$ first, ensuring that the semantic level of ${{\mathscr {F}}}\mathrm{{(x)}}$ is higher than that of $\mathrm{{x}}$. The *temp* is applied to implement the temperature annealing strategy, which is initialized to 30 and gradually decreases to 1 after 10 epochs. We verified the effect of *temp* on three models RA-ResNet18, RA-ResNet50, and RA-MobileNetV2 (1.0$\times$). The results are shown in Table 9.

**Table 9:** Effect of temperature annealing strategy.

Models	Temp	Top-1 Acc.	Top-5 Acc.
RA-ResNet18	–	71.0	90.0
	$\surd$	71.2	90.1
RA-ResNet50	–	77.4	93.8
	$\surd$	77.6	93.8
RA-MobileNetV2(1.0$\times$)	–	72.3	90.8
	$\surd$	72.5	90.9

The experimental results show that the temperature annealing strategy steadily improves the performance of the model. For instance, with the introduction of *temp*, the Top-1 accuracy of RA-ResNet18 and RA-ResNet50 are both improved by 0.2%. For the lightweight model RA-MobileNetV2(1.0$\times$), training with the temperature annealing strategy resulted in 0.2% and 0.1% improvement in Top-1 and Top-5 accuracy, respectively. In summary, the experimental results prove that the semantic gap between ${\mathscr {F}}\mathrm{{(x)}}$ and $\mathrm{{x}}$ is beneficial to the optimization of reverse attention.

**Table 10:** Effect of different training epochs on performance.

Method	Epochs	Top-1 Acc.	Method	Epochs	Top-1 Acc.
ResNet50	100	76.2	MobileNetV2 (1.0$\times$)	150	71.6
RA-ResNet50		77.6 ($\uparrow$1.4)	RA-MobileNetV2 (1.0$\times$)		72.5($\uparrow$0.9)
ResNet50	200	76.9	MobileNetV2 (1.0$\times$)	200	71.8
RA-ResNet50		78.4 ($\uparrow$1.5)	RA-MobileNetV2 (1.0$\times$)		72.9 ($\uparrow$1.1)
ResNet50	300	77.2	MobileNetV2 (1.0$\times$)	300	72.1
RA-ResNet50		78.5 ($\uparrow$1.3)	RA-MobileNetV2 (1.0$\times$)		73.1 ($\uparrow$1.0)

#### Training epochs

In order to explore the impact of different training epochs on the performance of the reverse attention mechanism, we further implemented experiments on ResNet50 and MobileNetV2 (1.0$\times$). As shown in Table 10, three different training epochs are set for each model. The results show that reverse attention can steadily improve the performance of ResNet50 under 100, 200 and 300 training epochs. Similar conclusions can be obtained by observing MobileNetV2 (1.0$\times$) at 150, 200, and 300 training epochs.

**Figure 4:**
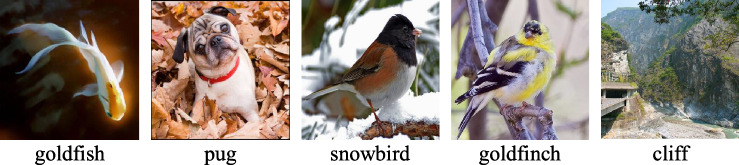
Sample images from the five classes of ImageNet-1 K.

**Figure 5:**
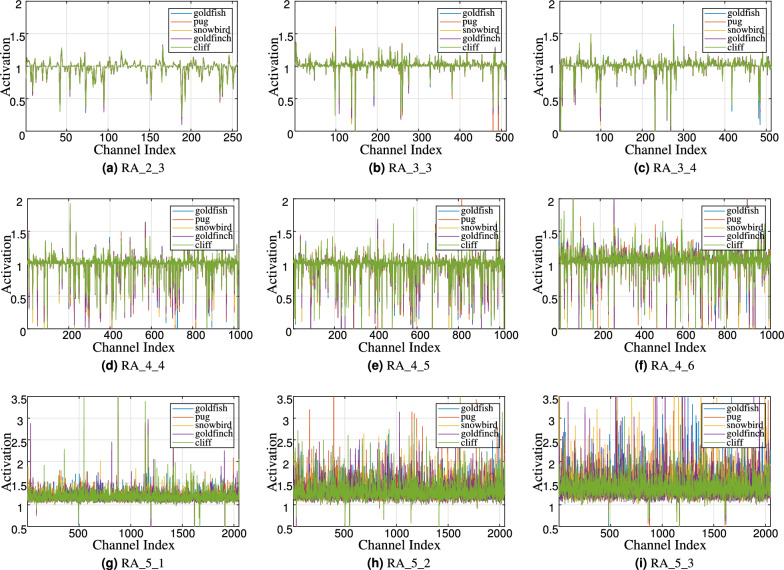
Activations induced by reverse attention at different depths in RA-ResNet-50 on ImageNet-1K. The nomenclature for each set of activations follows the RA_stageID_blockID scheme. For instance, the activation of the third block in the second stage can be identified as RA_2_3.

### RA activation

In this subsection, we show activations induced by reverse attention. As illustrated in Fig. 4, we select several test samples from the validation set of ImageNet-1K, encompassing five distinct categories: goldfish, pug, snowbird, goldfinch, and cliff. We implement experiments on RA-ResNet50 and report activations for different classes at different depths. The experimental results are reported in Fig. 5. The nomenclature for each set of activations follows the RA_stageID_blockID scheme. For instance, the activation of the third block in the second stage can be identified as RA_2_3. Meanwhile, to investigate the impact of depth, we present the RA activations for four stages of RA-ResNet50. According to Fig. 5, we can infer the following conclusions:

1. The activations are distributed on both sides of 1 (identity mapping). When the activation is greater than 1, the information is reinforced and transmitted to deeper layers. On the contrary, it is weakened or even blocked (activation close to 0). Consistent with the hypothesis, the network adaptively selects effective information while blocking the transmission of interference.
2. The impact of reverse attention (reinforcement or weakening) on information becomes more obvious as the depth increases. As an illustration, from Fig. 5a–i, the distribution of activations is increasingly spread out. In shallow layers, the model’s decision-making ability is hampered by the limitations of the semantic level and receptive field. Therefore, reverse attention performs close to identity mapping. With increasing depth, models benefit from richer information and larger receptive fields to enhance decision-making capabilities. Moreover, it is observed that the activations of the three blocks (RA_5_1, RA_5_2, RA_5_3) in stage 5 primarily serve as reinforcement. We conjecture that the model mainly suppresses interference in the fourth stage. Our assumption can be supported by the evidence presented in Fig. 5d–f.
3. The difference in activation among classes emerges gradually with increasing depth. For example, the distributions across different classes are very similar in the early layers of the model. In RA_2_3 and RA_3_4, the activations of the five classes are almost consistent. This suggests that the importance of features shared by different classes in the early stages. And they are distinguished in stage 4 and 5, especially in Fig. 5i (RA_5_3). These observations are consistent with previous research work^37^. That is, earlier layer features are generally more prevalent, while later layer features exhibit high levels of specificity.

In summary, we observe RA activation through instances of different classes. As expected, the reverse attention mechanism adaptively scales features. From the dimension of the channel, it enhances the effective information and blocks the transmission of interference.

## Conclusion

In this work, we propose a reverse attention mechanism, which utilizes high-level semantics to supervise low-level information. Meanwhile, based on reverse attention, we introduce a generalized residual learning framework, which is the RA architecture. Additionally, We implement the RA architecture with the proposed M-GRN and subsequently derive RA-Net from it. Compared to residual learning networks, RA-Net significantly improves performance with comparable model size and computational cost. This shows that the model’s performance can also be improved by the advantages of the architecture. Meanwhile, RA’s high-to-low guidance approach can also be applied to building frameworks in other areas.

## Data Availability

The data supporting the findings of this study are publicly available. The ImageNet-1k datasets are available at https://www.image-net.org/. The ADE20K datasets are available at http://groups.csail.mit.edu/vision/datasets/ADE20K/. The COCO datasets are available at https://cocodataset.org/.

## References

[1] He, K., Zhang, X., Ren, S. & Sun, J. Identity mappings in deep residual networks. In *European Conference on Computer Vision* 630–645 (2016).

[2] He, K., Zhang, X., Ren, S. & Sun, J. Deep residual learning for image recognition. In *IEEE Conference on Computer Vision and Pattern Recognition* 770–778 (2016).

[3] Yu, W. *et al.* Metaformer is actually what you need for vision. In *IEEE Conference on Computer Vision and Pattern Recognition* 10819–10829 (2022).

[4] Liu, Z. *et al.* A convnet for the 2020s. In *IEEE Conference on Computer Vision and Pattern Recognition* 11976–11986 (2022).

[5] Dosovitskiy, A. *et al.* An image is worth 16 $$\times$$ 16 words: Transformers for image recognition at scale. In *International Conference on Learning Representations* (2021).

[6] Ren S, He K, Girshick R, Sun J (2015). Faster r-cnn: Towards real-time object detection with region proposal networks. Adv. Neural Inf. Process. Syst..

[7] Han K (2022). A survey on vision transformer. IEEE Trans. Pattern Anal. Mach. Intell..

[8] Woo, S. *et al.* Convnext v2: Co-designing and scaling convnets with masked autoencoders. Preprint at http://arxiv.org/abs/2301.00808 (2023).

[9] Zhang, H., Hu, W. & Wang, X. Parc-net: Position aware circular convolution with merits from convnets and transformer. In *European Conference on Computer Vision* 613–630 (2022).

[10] Vaswani, A. *et al.* Scaling local self-attention for parameter efficient visual backbones. In *IEEE Conference on Computer Vision and Pattern Recognition* 12894–12904 (2021).

[11] Sandler, M., Howard, A., Zhu, M., Zhmoginov, A. & Chen, L.-C. Mobilenetv2: Inverted residuals and linear bottlenecks. In *IEEE Conference on Computer Vision and Pattern Recognition* 4510–4520 (2018).

[12] Zhang, X., Zhou, X., Lin, M. & Sun, J. Shufflenet: An extremely efficient convolutional neural network for mobile devices. In *IEEE Conference on Computer Vision and Pattern Recognition* 6848–6856 (2018).

[13] Tan, M. & Le, Q. Efficientnet: Rethinking model scaling for convolutional neural networks. In *International Conference on Machine Learning* 6105–6114 (2019).

[14] Liu, Z. *et al.* Swin transformer: Hierarchical vision transformer using shifted windows. In *IEEE International Conference on Computer Vision* 10012–10022 (2021).

[15] Wang, W. *et al.* Pyramid vision transformer: A versatile backbone for dense prediction without convolutions. In *IEEE International Conference on Computer Vision* 568–578 (2021).

[16] Chen, Y. *et al.* Mobile-former: Bridging mobilenet and transformer. In *IEEE Conference on Computer Vision and Pattern Recognition* 5270–5279 (2022).

[17] Xu, W., Xu, Y., Chang, T. & Tu, Z. Co-scale conv-attentional image transformers. In *IEEE International Conference on Computer Vision* 9981–9990 (2021).

[18] Mehta, S. & Rastegari, M. Mobilevit: Light-weight, general-purpose, and mobile-friendly vision transformer. In *International Conference on Learning Representations* (2021).

[19] Carion, N. *et al.* End-to-end object detection with transformers. In *European Conference on Computer Vision* 213–229 (2020).

[20] Hu, J., Shen, L. & Sun, G. Squeeze-and-excitation networks. In *IEEE Conference on Computer Vision and Pattern Recognition* 7132–7141 (2018).

[21] Woo, S., Park, J., Lee, J.-Y. & Kweon, I. S. Cbam: Convolutional block attention module. In *European Conference on Computer Vision* 3–19 (2018).

[22] Vaswani A (2017). Attention is all you need. Adv. Neural Inf. Process. Syst..

[23] Elfwing S, Uchibe E, Doya K (2018). Sigmoid-weighted linear units for neural network function approximation in reinforcement learning. Neural Netw..

[24] Memisevic R, Zach C, Pollefeys M, Hinton GE (2010). Gated softmax classification. Adv. Neural Inf. Process. Syst..

[25] Ioffe, S. & Szegedy, C. Batch normalization: Accelerating deep network training by reducing internal covariate shift. In *International Conference on Machine Learning* 448–456 (2015).

[26] Ba, J. L., Kiros, J. R. & Hinton, G. E. Layer normalization. Preprint at http://arxiv.org/abs/1607.06450 (2016).

[27] Chen, Y. *et al.* Dynamic convolution: Attention over convolution kernels. In *IEEE Conference on Computer Vision and Pattern Recognition* 11030–11039 (2020).

[28] Deng, J. *et al.* Imagenet: A large-scale hierarchical image database. In *IEEE Conference on Computer Vision and Pattern Recognition* 248–255 (2009).

[29] Polyak BT, Juditsky AB (1992). Acceleration of stochastic approximation by averaging. SIAM J. Control Optim..

[30] Paszke A (2019). Pytorch: An imperative style, high-performance deep learning library. Adv. Neural Inf. Process. Syst..

[31] Lin, T.-Y. *et al.* Microsoft coco: Common objects in context. In *European Conference on Computer Vision* 740–755 (2014).

[32] Zhou B (2019). Semantic understanding of scenes through the ade20k dataset. Int. J. Comput. Vis..

[33] Tian, Z., Shen, C., Chen, H. & He, T. Fcos: Fully convolutional one-stage object detection. In *IEEE Conference on Computer Vision and Pattern Recognition* 9627–9636 (2019).

[35] He, K., Gkioxari, G., Dollár, P. & Girshick, R. Mask r-cnn. In *IEEE International Conference on Computer Vision* 2961–2969 (2017).

[36] Xiao, T., Liu, Y., Zhou, B., Jiang, Y. & Sun, J. *Unified Perceptual Parsing for Scene Understanding* (2018).

[37] Lee, H., Grosse, R., Ranganath, R. & Ng, A. Y. Convolutional deep belief networks for scalable unsupervised learning of hierarchical representations. In *International Conference on Machine Learning* 609–616 (2009).

